# Height and health in late eighteenth-century England

**DOI:** 10.1080/00324728.2020.1823011

**Published:** 2020-09-29

**Authors:** Hannaliis Jaadla, Leigh Shaw-Taylor, Romola Davenport

**Affiliations:** 1 University of Cambridge; 2 Tallinn University

**Keywords:** socio-economic status, height, anthropometry, militia, health inequality, industrial revolution, eighteenth century, nutritional status, historical demography

## Abstract

Adult stature has become a widely used indicator of childhood nutritional status in historical populations and may provide insights into health inequalities that are not discernible in mortality rates. However, most pre-twentieth-century British data on heights suffer from selection biases. Here we present unique evidence on heights of adult males by occupation from an unbiased sample of adult males in Dorset in 1798–99. The mean height of fully grown (married) men was very similar to that of older military recruits, and our sample therefore confirms the taller stature of English males relative to males of other European countries in the same period. In contrast to previous evidence of negligible or U-shaped socio-economic gradients in mortality in this period, we found a fairly linear gradient in height by socio-economic status, that is similar in magnitude to class differences in adult height among English males born in the mid-twentieth century.

Supplementary material for this article is available at: https://doi.org/10.1080/00324728.2020.1823011

## Introduction

A major debate in demographic history is the extent to which health inequalities widened or narrowed during the demographic transition (Antonovsky 1967; Link and Phelan 1995; Woods and Williams 1995; Clouston et al. 2016). With respect to mortality, most studies report negligible differences in survival by wealth before the late nineteenth century (reviewed in Bengtsson and van Poppel 2011; Jaadla et al. 2020). In the English case, previous work has revealed only small gradients in survival by socio-economic status before the late nineteenth century. Adult life expectancies among male aristocrats only surpassed the national average in the mid-nineteenth century (Smith and Oeppen 2006). For children, recent work has demonstrated a U-shaped mortality curve with respect to socio-economic status in early childhood in the early nineteenth century (Jaadla et al. 2020). The children of labourers enjoyed high survival chances, despite their fathers being the poorest occupational group in the sample, and a social gradient in survival only emerged once this advantage was adjusted for. Even after adjustment the social gradient was small. In London socio-economic differences in survival in the first two years of life, when they could be measured, were also negligible in the period 1752–1812 (Davenport 2019). Even at the end of the nineteenth century, socio-economic gradients in infant mortality at the national level reflected mainly the differential sorting of occupational groups into residential areas with different mortality levels. Within these areas there was much less differentiation of mortality by paternal occupation (Reid 1997).

These results are surprising, because it might be expected that wealth conferred even greater advantages in historical settings, where food scarcity was common and welfare safety nets were minimal. One explanation offered for these findings is that exposure to disease was fairly uniform across social groups. Moreover, any greater access to medical care that the wealthy may have been able to buy did not improve outcomes because medical science was still poorly developed. However, disease outcomes can also depend on nutritional status, and we would expect a priori that wealthier groups would have enjoyed superior nutritional status to poorer groups. Addressing this paradox, Kunitz and Engerman (1992; following Kunitz 1983) argued that wealth may have conferred little survival advantage in traditional societies because some of the major causes of death in the past were lethal epidemic diseases against which nutritional status conferred little resistance.

Health is harder to measure than mortality, and in historical populations health status may not have moved in lockstep with trends in longevity (Kunitz 1987; Riley 1997). Therefore, it is possible that relatively small (or negligible) differences in mortality between socio-economic groups masked larger differences in health status in historical populations. The most commonly used proxy for health in historical populations is stature, and indeed, most studies that have examined heights by occupation or wealth report that height did vary with socio-economic status. In the English case, stature of military volunteers varied by occupation, and among cohorts born in the late eighteenth and early nineteenth centuries, teenage boys of the Marine Society were 10–22 centimetres (cm) shorter than the elite cadets of the Sandhurst military academy (Floud et al. 1990, p. 175; Komlos 2007). This difference is much larger than any reported in other European populations, and is often cited as evidence of large social inequalities in stature in Britain (Alter et al. 2004a; Komlos 2007; Steckel 2009; Meredith and Oxley 2014; Öberg 2014). However, existing data on stature in the English population are drawn from volunteer army and penal sources, and may suffer from a number of biases that make them unrepresentative of the general population, as discussed in the next section.

In this paper we describe our analysis of an apparently unique and unbiased source of data on English heights, which covers the adult male population of the county of Dorset for the years 1798–99. Where the source has survived, it provides height measures for all adult males aged 18–45 in every parish, together with information on occupation, marital status, number of children, and disability. This means that it provides representative data on height by occupational group, for comparison with existing military and penal sources from the same period. Our analyses confirmed previous claims that the average English male was tall compared with continental conscripts in the early nineteenth century. We also found a socio-economic gradient in height. However, socio-economic differences in height were relatively small, and consistent with those found in other nineteenth-century populations and in mid-twentieth-century British samples. That is, we found evidence for British exceptionalism with respect to height, but not with respect to inequality. More generally, the relatively small differences in stature between labourers and gentry in this period suggest either that all diets were deficient, regardless of wealth, or that exposure to disease was relatively undifferentiated by wealth or even inversely related to it. We discuss the limitations of our sample, in particular the lack of large town residents and the absence of age information.

## Background

Stature has been used extensively as an indicator of health and nutritional status in historical studies. This approach relies on the fact that while variations in stature between individuals are caused predominantly by genetic variation, differences in mean height between populations or subgroups generally reflect differences in environmental conditions during growth. A large number of factors can cause temporary or permanent stunting. These can be distinguished broadly as the influences of diet, disease, and work, and taken together they determine an individual’s ‘nutritional status’. These influences operate throughout the growth period, from gestation up until the late teens to mid-twenties, when growth ceases. Adult height therefore represents the complex integration of genetic and other influences on growth over 20 years or more.

Diet determines the gross amount of energy available for all processes, including growth, maintenance, temperature regulation, and work. However, in addition to sheer number of calories, the quality of diet also matters. Diets rich in proteins and dairy products may stimulate growth (Bogin 1999, pp. 276–81; Baten 2009). Deficiencies of macro- and micronutrients, such as calcium and vitamin D (from sunlight), can cause deficiency diseases (e.g. rickets) and stunting (Kirby 1995; Sharpe 2012). Importantly, dietary intake can also be compromised by infections. Sickness may reduce appetite, or cause loss of nutrients through vomiting and diarrhoea. However, acute illnesses are often followed by ‘catch-up’ growth, so may not cause stunting unless they are very frequent or lead to other complications (Eveleth and Tanner 1990, pp. 192–8). Of more importance are certain chronic infections. For example, sustained exposure to faecal matter is argued to cause gut enteropathy and malabsorption of food (Humphrey 2009). Parasitic worms can also disrupt gut function and reduce appetite (Hall et al. 2008).

Whether a given diet is sufficient for optimal growth also depends on the extent of other competing demands for energy. These include maintenance of bodily homeostasis, immune functions, reproduction, and work. Self-maintenance includes the regulation of body temperature, and the energy required for this varies with climate, season, housing, fuel, and clothing. The immune system also absorbs relatively large amounts of energy in responding to infection. Finally, energy requirements are strongly affected by activity levels. Children who work long hours at arduous tasks require more food than children who are more sedentary (Sharpe 2012).

A further complicating factor in assessing the determinants of variations in height is the potential for catch-up growth. In well-nourished and low-mortality populations, males now stop growing, on average, at around ages 17–20. However, under conditions of suboptimal nutrition or where growth has been delayed, individuals may continue to grow until their mid-twenties, and there is evidence for substantial growth after age 20 in some historical samples (Bogin 1999, pp. 90, 92; Alter et al. 2004b; Beekink and Kok 2017). Therefore variations in childhood conditions within populations may exert their greatest influence on *rates* of growth, and may have a smaller influence on final adult heights (Eveleth and Tanner 1990, pp. 192–8; Beekink and Kok 2017).

Given the complexity of the determinants of adult stature, and of health, it is unlikely that height provides a summary indicator of childhood health. However, to the extent that height reflects nutritional status in childhood, and that nutritional status during childhood can influence resistance to some infectious diseases, then height may reflect this aspect of childhood health.

**Figure 1:**
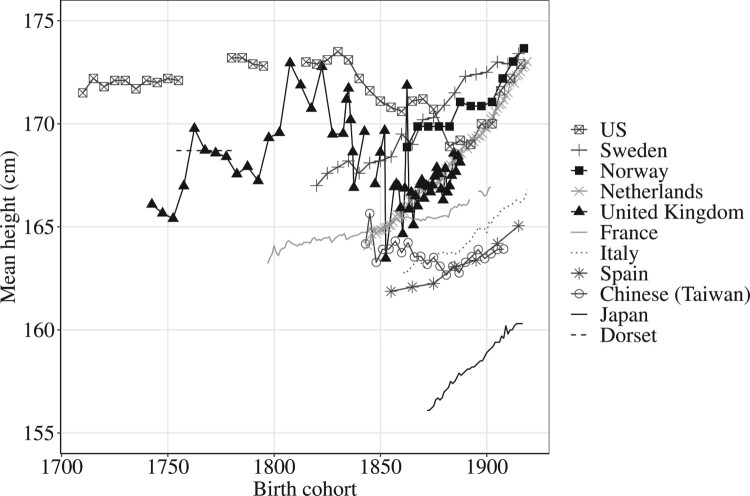
The development of mean heights of adult males in different populations, 1700–1920 *Source:* US, Table 9.A.1 in Fogel (1986); Sweden (Sandberg and Steckel 1997, p. 129); Norway, Appendix 2 in Hatton and Bray (2010); Netherlands (Drukker and Tassenaar 1997, pp. 356–57); United Kingdom (ages 24–29: Floud et al. 1990, pp. 148–49); France, Table 5B.1 in Weir (1997); Italy (Drukker and Tassenaar 1997, pp. 358–59); Spain (María-Dolores and Martínez-Carrión 2011, p. 35); Chinese (Taiwan), Table 3, model 2 in Olds (2003); Japan (Bassino 2006, p. 201); Dorset, authors’ own calculations.

Scholars have documented large differences in mean heights between populations in the past, and a general trend of increasing height with economic development. British and American men in particular appear to have been much taller than their European contemporaries in the early nineteenth century (Figure 1). However, the sources of evidence for stature differ between populations and over time, giving rise to debates about bias and comparability.

The data for continental European populations in Figure 1 derive from the military records of conscript armies, and are therefore fairly representative of the populations from which conscripts were drawn (after adjustment where necessary for the truncation of height distributions caused by the imposition of a minimum height requirement). However, in Britain and the United States (US), most height data for pre-twentieth-century populations derive from the records of volunteer armies and prisons (and records of sold or runaway slaves, in the US). This raises questions of representativeness. Military volunteers and criminals might not have been representative even of working-class men, and the composition of these groups may have changed over time. In particular, in periods or places where wages were high in other sectors, the army might have experienced more difficulty in recruiting men, and may have had to resort to recruiting shorter (and older) men, especially when demand for recruits was high. This effect is evident in the changing height requirements of military services over time (Floud et al. 1990, pp. 60, 65).

Bodenhorn et al. (2017) argued that selection biases arising from labour market effects explain the unusual trends in height in Anglophone sources across the nineteenth century. While average heights of continental military conscripts generally exhibited a fairly monotonic rise in height across the nineteenth century, English and American males born in the middle decades of the nineteenth centuries were apparently shorter than previous or later generations (Figure 1), despite the economic growth and rising incomes that occurred in this period (Broadberry et al. 2015). It remains contested whether these anomalous patterns reflect a trade-off of health and stature for wealth in the early phase of urbanization and industrialization, or reflect shortcomings of Anglophone sources of height data (Bodenhorn et al. 2017). This study does not contribute to the debate regarding trends in height in the English population; however, it does provide a point measure of height for a socially representative sample, something unique for England before the twentieth century.

**Figure 2:**
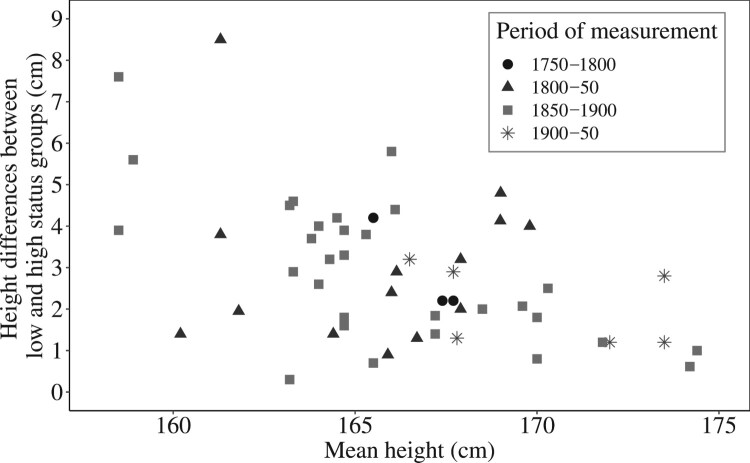
Social inequalities in height over time for cohorts born before the twentieth century, various populations *Sources:* Komlos 1987, 1990, 1994; Floud et al. 1990; Twarog 1997; Baten & Murray 2000; A’Hearn 2003; Alter et al. 2004a; Cranfield and Inwood 2007; Heyberger 2007; Martínez-Carrión and Moreno-Lázaro 2007; Cinnirella 2008; Baten et al. 2009; Lantzsch and Schuster 2009; Schoch et al. 2012; Manfredini et al. 2013; Sunder 2013; Ayuda and Puche-Gil 2014; Öberg 2014; López-Alonso and Vélez-Grajales 2015; Bailey et al. 2016; Beekink and Kok 2017; Mazzoni et al. 2017; Koepke et al. 2018; Llorca-Jaña et al. 2018; Quanjer and Kok 2019; Tassenaar 2019. See Table A1, supplementary material, for full list of studies and the social class groups compared in each.

There is a very large literature on the interpretation of heights in historical populations. However, relatively few studies contain information on height by socio-economic status. Studies of late-twentieth-century populations generally indicate persistent social inequalities in height (e.g. Li and Power 2004; Singh-Manoux et al. 2010; Schoch et al. 2012; Lopuszanska-Dawid et al. 2020), and socio-economic gradients in child and adult heights have also been reported in historical studies. Figure 2 presents data from studies that reported heights of adult males in cohorts born before the twentieth century, for representative samples comprising all status groups. The gradients reported are generally in the order of 1–5 cm between poor and wealthy/well-fed groups, and they appear to have attenuated as populations grew taller (Figure 2).

The studies included in Figure 2 report height differentials in a variety of ways, and cannot be considered strictly comparable. Some of the data refer to individual towns or regions, and some measures represent raw differences in average heights, while others have been adjusted for various factors. In any case, basic differences in social organization between the societies being compared make it impossible to compare strictly equivalent social status groups. Readers are referred to Table A1 in the supplementary material for the sources of data and for potential alternative measures of social class differences in heights contained in these sources. Notwithstanding these issues of comparability, the largest difference in stature reported in Figure 2 is of 8.5 cm, between urban labourers and students in eastern Belgium in 1816–49 (Alter et al. 2004a). These data can be compared with the 10–22 cm difference between the very deprived boys of the Marine Society, and the mainly aristocratic cadets of Sandhurst military academy, measured at ages 13–16 in early-nineteenth-century England (Floud et al. 1990, pp. 163–78; Komlos 2007).

The huge differences in height between Marine Society and Sandhurst boys are not reported in Figure 2, because they do not meet the criteria for inclusion, that is, they are not drawn from a representative sample covering a wide social range. The Marine Society recruited poor boys from London who were unable to obtain any other kind of employment or apprenticeship. Some of them were vagrants, and all were very deprived (Floud et al. 1990, pp. 55–6). In addition, most would have been exposed during gestation and childhood to the very severe disease environment that prevailed in late-eighteenth-century London. In contrast, Sandhurst was established in 1802 to train the sons of dead or impoverished military officers, but was almost immediately expanded to admit fee-paying sons of elite families drawn from across Britain (Mockler-Ferryman 1900, pp. 10–11). Not only were these boys extremely privileged, but the distribution of their heights suggested to Komlos that (high) minimum height requirements were either enforced or self-imposed on potential cadets (Komlos 2007). Therefore, these two groups probably represented the very extremes of deprivation and disease exposure on the one hand and privilege on the other.

Floud et al. also compared the heights of army recruits by their stated occupation before enlistment. In contrast to comparisons of Marine Society and Sandhurst boys, they found only small differences in height between military recruits from different occupations (who were born c. 1815): 1.3 cm between the tallest, in commercial occupations, and the shortest, servants (in adjusted models: Floud et al. 1990, pp. 203, 217–23). These small differences in stature may reflect the limited social range of recruits to the British military. However, this also draws attention to the extreme nature of the comparison between Marine Society and Sandhurst boys.

For the study reported in this paper, we used an apparently unique source of heights data for the county of Dorset in 1798–99 to test whether socio-economic differences in height were indeed exceptionally large in the English population in this period.

## Data and methods

The Dorset Militia Ballot Lists include the full height and social distributions of adult males in late-eighteenth-century Dorset. The Militia Act of 1757 introduced county-level quotas for militia service. Parish constables were required to draw up full lists of adult males, and then these men (with some exemptions) were subjected to a ballot to choose those required to serve. Reasons for exemption included short stature (under 5 feet 4 in.), and, for poor men, having three or more children aged under ten. Importantly, the militia ballot lists (lists of men potentially liable to serve) included all men in the parish within a stipulated age range. These lists initially recorded men aged 18–50 annually, excluding those who were peers, clergy, teachers, and apprentices. However, in 1758 the Act was amended to include all names, and in 1762 the upper age limit was lowered to 45 (Gibson and Medlycott 2013). There was no requirement to record heights at the balloting stage. However, heights were widely recorded in Dorset during the 1790s (Medlycott 1999a, 1999b).

Unfortunately, the surviving militia ballot lists for Dorset are only fragments of the full set of annually compiled lists, which were mostly destroyed by damp storage conditions around 1900. We used surviving lists for the years 1798 and 1799 because heights and family information appeared to be fairly complete for these years (Table 1). These lists included information on name, occupation, different health impairments, current or previous military service, place of residence or enumeration, and height. The lists also recorded information on whether listed men were married and the number of children they had. It was probably intended that the lists should record children under ten only, but Medlycott (1999a, 1999b) argued that it was very likely that for some men all of their children were counted. In support of this, the lists included 117 men with seven or more children.

**Table 1:** Number of observations in Medlycott’s Dorset Militia Ballot Lists, and percentage including information about height or family (wife/children), 1798–99

	Year	
	1798	1799
Observations	5,657	2,978
Percentage with height	96.34	96.71
Percentage listed with wife, children, or both	44.86	41.57
Mean height in cm (SD)	168.7 (5.6)	168.9 (5.2)

*Notes:* Observations exclude entries for tithingmen and constables without heights, because these men were signatories to the lists but were not themselves listed. Lists of owners of mills and ovens in 1798 are also excluded. SD refers to the standard deviation.

*Source:* Medlycott 1999a, 1999b.

**Figure 3:**
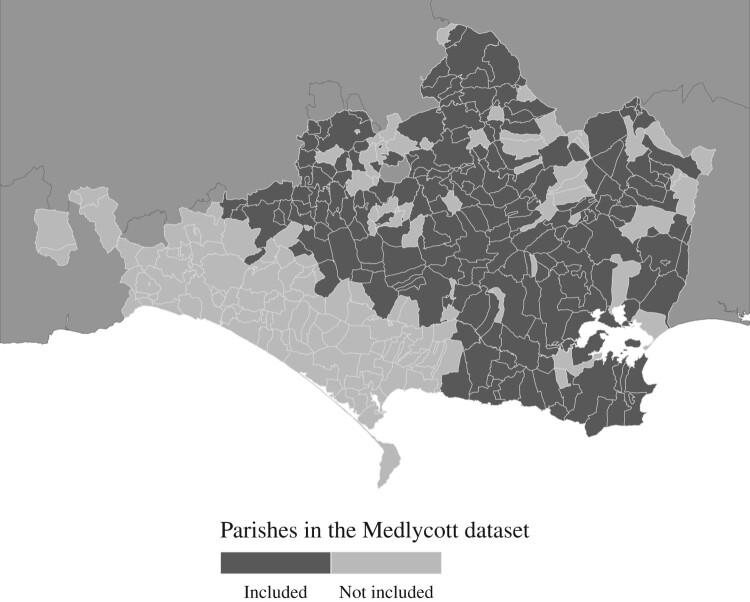
The coverage of parishes in the Medlycott data set, Dorset, 1798–99 *Source:* Medlycott 1999a, 1999b.

The data set contained 8,330 observations, with reported heights from 227 parishes, and included cohorts born from 1754–55 to 1780–81. Recruitment in Dorset was organized into sub-parochial units called tithings, but for our analysis we grouped different tithing listings into parishes. Some of the parishes listed men in both 1798 and 1799. In order to avoid duplicate entries for the same individuals, we included each parish only once, leaving us with 6,753 observations with information about height. Our strategy was to remove the year in which the parish with repeat measurements had the smaller number of observations. Where the number of observations was equal in both years (Frome St. Quintin, Hampreston, West Stour, and Winterborne Zelston), then we included only the year 1799. In models (not shown) that used the excluded data, all results were in the same direction; only the magnitudes of coefficients were slightly different. In addition, inter-year comparisons of mean heights in parishes that recorded heights in more than one year (for the years 1796, 1798, and 1799) indicated significant differences in only a small minority of parishes, and the direction of these differences was not consistent. Therefore, there was no indication of a systematic bias in the measurement of heights between years, or of selection effects. Figure 3 demonstrates the geographical coverage of Medlycott’s lists for the years 1798–99. Data for much of coastal West Dorset and the town of Weymouth are missing.

**Figure 4:**
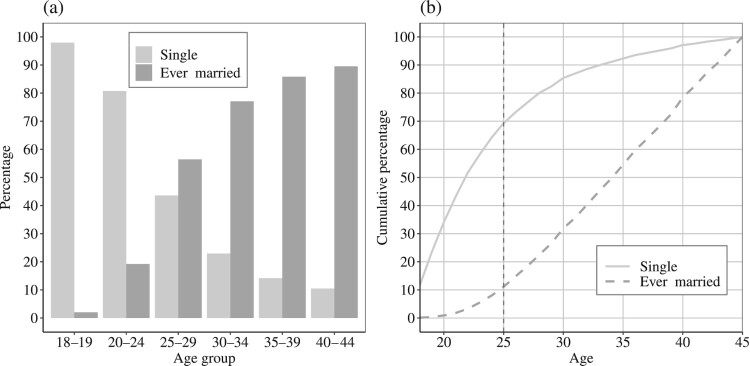
(a) Percentages of men aged 18–45 single and ever-married in each age group and (b) Cumulative percentages of single men and ever-married men under given ages in Dorset, 1851 *Note:* The vertical dashed line in (b) indicates the percentages of single/married men who were aged under 25. *Source:* Schürer et al. 2016.

Unfortunately, the Dorset Militia Ballot Lists do not include information about the exact birth year or age for men listed in these ballots. The age range of men in our sample was 18–45, and therefore some of the younger men in our sample may not have reached full adult height at the time of measurement. For that reason, we distinguished between married and single men, assuming that married men were generally older and had reached their adult height. This assumption is supported by some of the earliest evidence for age patterns in marital status for men living in Dorset, available from individual-level data from the 1851 Census (Schürer et al. 2016). The proportions married in the mid-nineteenth century show that almost no men were married before age 20 (Figure 4, left-hand panel). In the age group 25–29 more than 55 per cent of men were married and this increased continuously with age. The cumulative distribution (right-hand panel) shows that 70 per cent of all single men and only 10 per cent of married men aged 18–45 were younger than 25. The median age of single men was 22 years, and for ever-married men it was 34. We can assume that fairly similar marriage patterns held for the late eighteenth century. On the assumption that final adult height was attained by age 25, then these marriage patterns imply that almost all married men in our sample had already attained their full height, but that many unmarried men had not. At the other end of the age distribution, adults also begin to lose height as they age; however, the upper age limit, 45 years, was low enough that age-induced shrinkage probably did not affect our sample.

An obvious question is whether males were selected into marriage only on the basis of age, or whether height was also a factor, for example if taller men were more likely to marry. Unmarried men were only 1 cm shorter, on average, than married men (Table 2). Estimates of mean growth between ages 18 and 24 are as much as 3–5 cm in previous studies (Alter et al. 2004b; Beekink and Kok 2017), so the difference between single and married men in our study was within the range expected if it were due simply to the younger average age of single men. However, it is also possible that selection into marriage was more stringent for some occupations, and this could distort our measures of socio-economic differences in height by marital status. This issue is addressed more fully in Table A2 in the supplementary material, which demonstrates that differences in height and in the prevalence of disabilities by marital status did not vary markedly by socio-economic status, for the numerically largest occupations. Table A2 also reports singulate mean age at marriage for the same occupations in 1851—again, there was no obvious socio-economic patterning of age at marriage.

**Table 2:** Descriptive statistics for Dorset sample, 1798–99

	Full sample	Single men	Married men
Mean height in cm (SD)	168.7 (5.4)	168.3 (5.6)	169.3 (5.1)
Mean relative wealth (SD)	23.8 (21.6)	22.8 (21.1)	25.0 (22.2)
HISCLASS (percentage)			
Elite	3.0	2.5	3.6
Lower middle class	4.9	5.1	4.7
Skilled workers	38.5	42.4	33.4
Farmers and yeomen	8.8	9.7	7.6
Unskilled workers	44.8	40.3	50.7
Parish type (percentage)—urban	23.7	24.5	22.7
Health status (percentage)			
No health conditions	93.2	94.1	92.1
Physical injuries	3.9	3.3	4.8
Deaf, dumb, or blind	1.4	1.2	1.6
Other	1.5	1.4	1.5
Mean number of children (SD)	1.0 (1.7)	–	2.3 (1.9)
Year of ballot (percentage)			
1798	70.3	68.1	73.1
1799	29.7	31.9	26.9
Number of observations	6,753	3,810	2,943

*Notes:* Parishes categorized as urban in our sample were: Abbotsbury, Beaminster, Blandford Forum, Bridport, Cerne Abbas, Corfe Castle, Cranborne, Dorchester, Evershot, Lyme Regis, Milton Abbas, Poole, Shaftesbury, Sherborne, Stalbridge, Sturminster Newton, Wareham, Weymouth, and Wimborne Minster. SD refers to the standard deviation.

*Source:* Medlycott 1999a, 1999b; authors’ own estimation.

**Figure 5:**
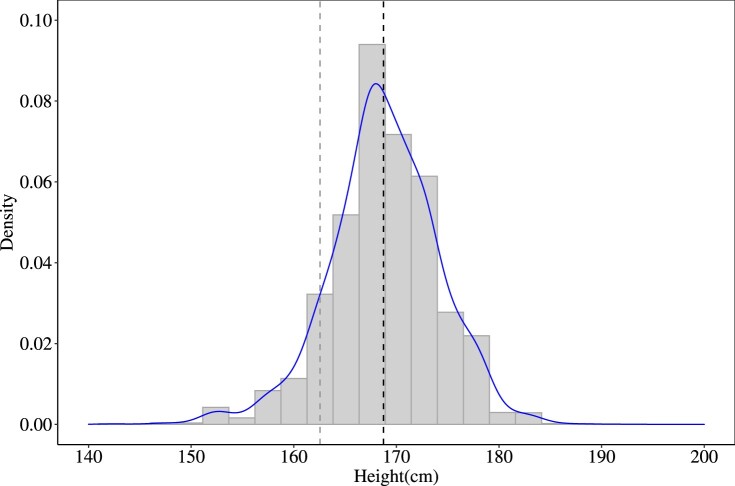
Distribution of heights (cm) in the Medlycott data set, 1798–99 *Notes:* Dashed lines indicate, from left, the minimum height requirement for militia service, and the mean height of the sample. *Source:* As for Figure 3.

In order to investigate the relationship between wealth and height, we estimated Ordinary Least Squares (OLS) models where height (in cm) was the dependent variable. Heights were reported in full inches in the militia ballot lists, and we do not know how the measurements were rounded (e.g. to the nearest inch). Where we could identify the same man in lists in 1798 and 1799, the difference in height between the two dates was 0–1 inch in 77 per cent of cases, suggesting consistency in measurement over time, at least within parishes. While there were clearly some errors (assuming that we had correctly identified the same man in both years), the spread of errors followed a normal distribution, suggesting that any errors were random. The average height of all men in our subsample of 1798–99 was 168.7 cm. This is very similar to the estimated average height for English military recruits from the same birth cohorts at ages 24–29 reported by Floud et al. (1990, p. 148). Figure 5 shows the overall distribution of heights. The distribution is not completely normal, possibly because it includes men from quite a wide age range (18–45), where some had not yet reached adult height (A’Hearn et al. 2009). However, as expected, there was no evidence of any truncation that would suggest the operation of a minimum height requirement.

Our main variable of interest was the socio-economic status of individual men as measured by their occupation, which we interpreted both as an indicator of status of the individual and of his family of origin. The key assumption was that the occupations reported by men in militia lists mirrored the socio-economic background they were raised in. This is a relatively common approach in anthropometric literature, and assumes a low degree of intergenerational mobility (Cinnirella 2008; Lantzsch and Schuster 2009). The limited evidence for intergenerational and lifetime occupational mobility in England in this period supports this assumption (Clark and Cummins 2014). However, it is important to recognize that any apparent association between height and occupation will reflect not only differences in living conditions during childhood, but also other confounding effects of childhood socio-economic status, and possibly height-based selection into occupation (Lantzsch and Schuster 2009; see also ‘Discussion’).

Previous studies analysing socio-economic differences in historical contexts have used a number of different social stratification schemes based on ranking historical occupations. For research in this paper, we took two approaches. First, we used a commonly applied HISCLASS scheme based on five social classes (Van Leeuwen and Maas 2011). These were: (1) elite; (2) lower middle class—clerks, merchants, and dealers; (3) skilled workers—different types of makers, smiths, and weavers; (4) farmers and yeomen (including sons of farmers); and (5) unskilled workers—agricultural and general labourers. Notably, farmers and other agriculturists are accorded a relatively low status in HISCLASS. However, southern England, including Dorset, was characterized in this period by capital-intensive agriculture and a wide social gulf between farmers and their labourer employees (Shaw-Taylor 2012). Our sample exemplified this pattern of a highly proletarianized agricultural workforce: the ratio of wage labourers to farmers in rural parishes was 4.6:1. Farmers in Dorset in this period were also 50 times more likely to leave a probate record (of movable wealth at death) than labourers (as described shortly). The poor fit of HISCLASS to English society in this period is discussed further in Jaadla et al. (2020).

As our second measure of socio-economic status, we used a continuous measure of the relative wealth of an occupation, derived from English parish registers and probate records (Keibek 2017). Parish registers recorded the occupation of fathers at baptism (comprehensively after 1812), but they provide incomplete national coverage. Probate records survive for the whole of England and they record occupation and movable wealth at death, but are socially selective. Keibek combined these two measures, where they were available for the same parish, to create a ratio measure of the frequency of an occupation appearing in probate records compared with its frequency in baptism registers covering the same area. The occupation ratios were then indexed against the ratio for farmers. For example:

(1) $$\frac{\text{number}\;\;\text{of}\;`>;\text{dealers}\;\text{in}\;{\text{drink}}^{\prime }\;\text{in}\;\text{parish}\;\text{register}}{\text{number}\;\text{of}\;`>;\text{dealers}\;\text{in}\;{\text{drink}}^{\prime }\;\text{in}\;\text{probate}\;\text{records}\;}=\frac{68}{4}=17$$

(2) $$\frac{\text{number}\;\text{of}\;`>;{\text{farmers}}^{\prime }\;\text{in}\;\text{parish}\;\text{register}}{\text{number}\;\text{of}\;`>;{\text{farmers}}^{\prime }\;\text{in}\;\text{probate}\;\text{records}}=\frac{768}{74}=10.4$$

(3) $$\frac{`>;\text{dealers}\;\text{in}\;{\text{drink}}^{\prime }\;\text{ratio}}{`>;{\text{farmers}}^{\prime }\;\text{ratio}}=\frac{17}{10.4}=1.6$$

The relative wealth measure or occupational multiplier for ‘dealers in drink’ is 1.6 in this example, meaning that they were 1.6 times less likely to leave a probate record than farmers. The propensity to leave a probate record was strongly correlated with the median value of the probated goods by occupation (Keibek 2017, p. 93). Our measures were based on ratios calculated for Dorset in 1813–20. The value of occupational multipliers in our sample ranged from 0.29 for esquires and gentlemen to 49.46 for labourers, the occupational group that was least likely to leave a probate record.

Table 2 reports the distribution of socio-economic measures and other variables of interest. The majority of men in our sample were skilled workers (38.5 per cent) or unskilled workers (44.8 per cent). Only 3 per cent of men belonged among the elite. Our sample of men appears to be representative of the occupational structure for men in the county. Table 3 provides comparative estimates from a similar time period for the county of Dorset and for England (Keibek 2017). The distribution of occupations by sector in the Dorset Militia Ballot Lists (which were weighted towards the east of the county) was similar to the estimated distribution for the whole of Dorset in 1801, except that a somewhat larger proportion of men were listed in the tertiary sector in our data set. Compared with the national average, our sample included many more men in the primary sector and fewer in the secondary sector, and this reflects the more agricultural economy of Dorset compared with the national average in 1801.

**Table 3:** Male occupational structure (percentage) by sector in Dorset and England, around 1800

	Our sample: Dorset militia 1798–99*	Dorset 1801**	England 1801**
Primary sector	53.1	55.0	42.6
Secondary sector	29.9	35.0	42.0
Tertiary sector	17.0	10.0	15.4

*Source:* *authors’ own estimation; **Keibek 2017 (derived from probate inventories for 1801, using adjustment ratios for social bias calculated for 1813–20).

In order to estimate the influence of socio-economic status on height, our models included an additional set of control variables. Urban or rural place of residence was included as a proxy for significant differences in local disease environments, as negative effects of urban living might have had considerable influence on nutritional status. In our sample, about one in four of the men was listed in an urban parish. As we had no information about place of birth or place of residence in childhood, our analysis assumed that men’s place of residence at the time of being listed also reflected their early life conditions. This will of course not be correct for all of them. In particular, it is likely that many men listed in urban centres were in fact migrants, who had grown up elsewhere and migrated as teenagers or young adults. It is also possible that migrants to towns were positively selected for height (Humphries and Leunig 2009b).

Table 2 shows that only modest proportions of men mentioned any medical or health conditions in the ballot list: 3.9 per cent reported physical injuries, 1.4 per cent were deaf, dumb, or blind, and another 1.5 per cent included other mentions of sickness (asthma, consumption, mental health issues, etc.). Married men reported a higher prevalence of disabilities, and this probably reflects their higher average age and the age-dependent accumulation of conditions. It is important to bear in mind that some of the disabilities reported would have occurred in childhood or in utero, and some of these may have been associated with growth stunting. However, other disabilities would have been acquired as a result of accidents or infections at ages after adult height was attained.

The models also included a dummy variable for the year of balloting, to control for possible differences in the ballot system or enlistment by year.

## Results

**Figure 6:**
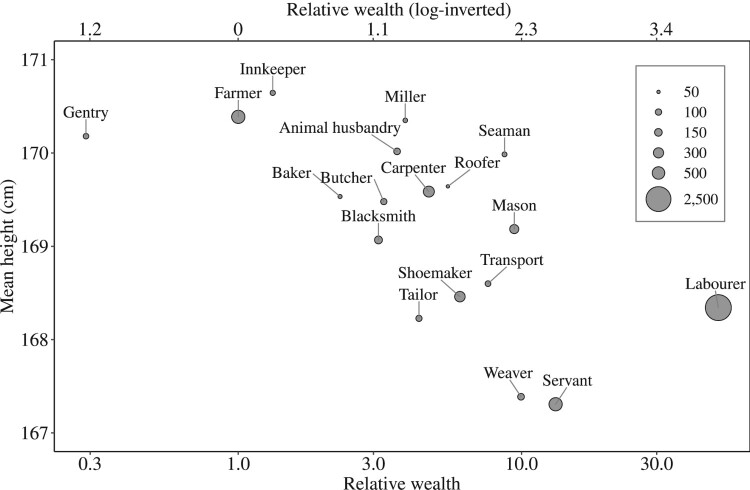
Mean height (cm) by relative wealth of occupational group, 1798–99 *Note:* The lower x-axis expresses wealth as a standardized ratio that is inversely proportional to wealth, while the upper x-axis expresses wealth in its (logged) inverse form that is directly proportional to relative wealth. *Source:* As for Figure 3.

Figure 6 shows the distribution of larger occupational groups in our data set (those with over 50 observations) by mean height and their relative wealth measure. The wealth measure is expressed as a standardized ratio (which is inversely proportional to wealth) on the lower x-axis, and in its inverse form on the upper x-axis (where the measure is directly proportional to relative wealth, as used in our regression modelling). Figure 6 shows a relatively clear wealth gradient in height. The occupations at the upper end of the wealth distribution (higher wealth) demonstrated a height advantage compared with occupations at the other end of the scale (lower relative wealth). On average, the farmers and the gentry in Dorset were taller than the weavers, servants, and labourers. However, these differences in stature were only of around 2–3 cm between the top and the bottom of the social ladder, a possible indication of a relatively low level of inequality in childhood nutritional status in late-eighteenth-century Dorset.

Tables 4 and 5 present our results using Keibek’s relative wealth measure in stepwise regression models. In all regression models, the dependent variable was height in cm and, for simplicity of interpretation, the relative wealth measure was inversed, log-transformed, and centred (by subtraction of the mean) in the models.

**Table 4:** OLS regression results (dependent variable height in cm): men in Dorset Militia Ballot Lists, 1798–99

	Model 1	Model 2	Model 3	Model 4	Model 5	Model 6	Model 7
*(Intercept)*	168.7	168.3	168.3	168.3	168.8	168.4	168.4
Relative wealth	0.43***	–	0.44***	0.42***	–	–	0.42***
	*(0.05)*	–	*(0.05)*	*(0.06)*	–	–	*(0*.*06)*
Marital status—married	–	0.97***	1.01***	1.01***	–	0.93***	0.96***
	–	*(0.13)*	*(0.13)*	*(0.13)*	–	*(0*.*14)*	*(0*.*14)*
Relative wealth × married	–	–	–	0.03	–	–	0.04
	–	–	–	*(0.09)*	–	–	*(0*.*09)*
Health status							
Physical injuries	–	–	–	–	−1.20***	−2.12***	−2.05***
	–	–	–	–	*(0.34)*	*(0*.*49)*	*(0*.*49)*
Deaf, dumb, or blind	–	–	–	–	−0.61	−1.07	−0.87
	–	–	–	–	*(0.57)*	*(0*.*80)*	*(0*.*79)*
Other	–	–	–	–	−0.90†	−0.64	−0.58
	–	–	–	–	*(0.54)*	*(0*.*73)*	*(0*.*73)*
Health status × marital status							
Physical injuries × married	–	–	–	–	–	1.57*	1.68*
	–	–	–	–	–	*(0*.*67)*	*(0*.*67)*
Deaf, dumb, or blind × married	–	–	–	–	–	0.79	0.79
	–	–	–	–	–	*(1*.*13)*	*(1*.*12)*
Other × married	–	–	–	–	–	−0.62	−0.63
	–	–	–	–	–	*(1*.*09)*	*(1*.*08)*
Year of ballot—1799	0.05	0.10	0.11	0.11	0.02	0.08	0.09
	*(0.14)*	*(0.14)*	*(0.14)*	*(0.14)*	*(0.14)*	*(0*.*14)*	*(0*.*14)*
*R*^2^	0.01	0.01	0.02	0.02	0.00	0.01	0.02
Number of observations	6,753	6,753	6,753	6,753	6,753	6,753	6,753

***p < 0.001; **p < 0.01; *p < 0.05; †*p* < 0.1.

*Notes:* Figures in brackets are standard deviations. Reference categories are unmarried (for marital status), no disability (for health status), and 1798 (for year of ballot).

*Source:* As for Table 2.

**Table 5:** OLS regression results (dependent variable height in cm) including urban residence: single and married men in Dorset, 1798–99

	*Single men*			*Married men*		
	Model 1	Model 2	Model 3	Model 4	Model 5	Model 6
(Intercept)	168.3	168.3	168.4	169.4	169.5	169.5
Relative wealth	–	0.45***	0.44***	–	0.50***	0.44***
	–	*(0.07)*	*(0*.*07)*	–	*(0.07)*	*(0*.*07)*
Parish type—urban	−0.33	−0.50*	−0.54*	−0.37	−0.67**	−0.78**
	*(0.20)*	*(0.21)*	*(0*.*21)*	*(0.23)*	*(0.23)*	*(0*.*24)*
Relative wealth × urban	–	–	−0.03	–	–	0.30†
	–	–	*(0*.*16)*	–	–	*(0*.*17)*
Health status						
Physical injuries	–	–	−2.10***	–	–	−0.42
	–	–	*(0*.*50)*	–	–	*(0*.*44)*
Deaf, dumb, or blind	–	–	−0.93	–	–	−0.10
	–	–	*(0*.*82)*	–	–	*(0*.*76)*
Other	–	–	−0.53	–	–	−1.20
	–	–	*(0*.*75)*	–	–	*(0*.*76)*
Year of ballot—1799	0.28	0.36†	0.34†	−0.06	−0.10	−0.09
	*(0.19)*	*(0.19)*	*(0*.*19)*	*(0.22)*	*(0.21)*	*(0*.*21)*
*R*^2^	0.001	0.01	0.02	0.001	0.02	0.02
Number of observations	3,810	3,810	3,810	2,943	2,943	2,943

***p < 0.001; **p < 0.01; *p < 0.05; †*p* < 0.1.

*Notes:* Figures in brackets are standard deviations. Reference categories are rural (for parish type), no disability (for health status), and 1798 (for year of ballot).

*Source:* As for Table 2.

We found clear socio-economic differences in height in the late eighteenth century. In Table 4, model 1, which included only relative wealth and a control variable for year of ballot, wealth was positively associated with height (a one-unit increase in wealth was associated with a 4.3 millimetre (mm) increase in height). The size and strength of the effect of wealth on height remained virtually unchanged after controlling for other possible determinants including marital status and disability (Table 4, models 3, 4, and 7).

Marital status, which we took as a proxy for the attainment of final adult height, was also associated with taller stature. In model 2, which included only marital status and height, and a control variable for year of ballot, being married was associated with a height advantage of 9.7 mm, and this advantage was virtually unchanged when wealth was included in the model (Model 3). To test whether the positive association between height and wealth differed by marital status, model 4 included an interaction between wealth and marital status. The interaction term was small and statistically insignificant, indicating that socio-economic gradients in height were similar for unmarried (and mainly younger) men and married (mainly fully grown) men (see also Figure A1 in the supplementary material). These results imply that a one-unit increase in relative wealth (equivalent to the difference between being a butcher or a surgeon, or between a shoemaker and a schoolmaster) meant a 4.2 mm increase in height for single men and a 4.5 mm (4.2 + 0.3) increase in height for married men.

Health status, and especially physical injuries (which included lameness and loss of digits or limbs), had a large effect on height (Table 4, model 5). The negative effect of physical injuries on height was largely confined to single men, as seen in model 6. Those unmarried men who reported any physical injuries at the time of being listed were on average >2 cm shorter (21.2 mm) than single men with no health conditions, whereas married men with physical injuries were only half a centimetre shorter (−21.2 + 15.7 = −5.5 mm) than their uninjured peers (see also Table 5, models 3 and 6). This suggests that some physical disabilities were associated with stunting in childhood, and that these may also have reduced the chances of marriage. It seems likely that it was the disability itself, rather than stunting, that reduced the chances of marriage, since there is no evidence that unmarried men in general were unmarried on account of short stature (as discussed in the ‘Data and Methods’ section). The finding also suggests that most of the disabilities reported by married men (where the prevalence of disability was greater) were either acquired in adulthood, or involved childhood trauma that did not result in stunting or reduced marriage chances. Model 7 (Table 4) included relative wealth in a full model, and confirmed that the effect of wealth remained very consistent after adjusting for marital status and disabilities.

Table 5 explores the associations between urban residence and height. To simplify the interpretation of the models, we stratified by marital status, which we used as a proxy for whether men were likely to have attained full adult height. Men listed in urban parishes were shorter than the men listed in rural parishes—the estimated coefficients imply that single men in urban parishes were 5.0 mm shorter and married men 6.7 mm shorter (after adjustment for wealth) (Table 5, models 2 and 5). These results are perhaps surprising, because the towns in our sample were small (the largest, Poole, contained a population of 4,761 in the 1801 Census). Humphries and Leunig (2009a, 2009b) found no effect of being born in a small urban centre (population <8,000) on heights of merchant seamen born in the early decades of the nineteenth century, and only small effects of being born in London (a reduction of <2 cm). Cinnirella (2008) also reported that British military recruits from large towns and even London were not shorter than rural recruits, for cohorts born before 1800 (although birth in London was associated with a 2.4 cm deficit in stature in cohorts born after 1800).

Continental studies of conscripts, where selection biases should be minimal, have found mixed effects of urbanization. In eastern Belgium, French Alsace, Württemberg, and Bavaria there was no evidence of an urban height penalty before the 1840s (Twarog 1997; Alter et al. 2004b; Heyberger 2007; Lantzsch and Schuster 2009). To some extent the lack of an urban disadvantage in these samples reflects the poverty of the rural areas from which recruits were drawn in this period (Alter et al. 2004b). Heyberger (2014) argued in the French case that the stunting effect of urban centres was masked in France by the greater average wealth of urban dwellers, and an urban height penalty became evident once occupational differences in height were controlled for. This was also the case in our study: the urban height penalty became statistically significant only in multivariate models that included wealth or occupational ranking (in Table 5, compare bivariate models 1 and 4 with the multivariate models).

**Figure 7:**
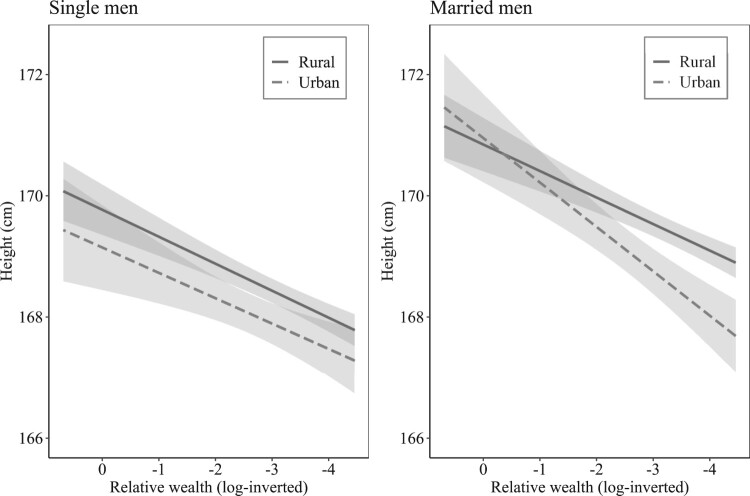
Predicted interaction effects of wealth and parish type on height, by marital status, Dorset Militia Ballot Lists, 1798–99 *Note:* Shaded areas represent 95 per cent confidence intervals. *Source:* As for Figure 3.

To explore further how socio-economic status and urban residence influenced stature, we estimated models that included an interaction between wealth and urban residence (Table 5, models 3 and 6). In these models the main effects remained virtually the same; however, the interaction effects indicated that the disadvantages of urban living on the stature of married men mainly affected the lower end of social distribution (Figure 7). Married labourers in towns were on average 1 cm shorter than labourers residing in more rural parishes. However, the stature of higher-status males (farmers and gentry) was similar in rural and urban parishes.

These results present a puzzle. Why did the urban penalty apparently fall mainly on poorer occupational groups, and on married men within these groups? The urban penalty is usually considered to be associated with disease, and in the eighteenth century infant and child mortality rates were much higher even in small towns than in rural areas (Wrigley et al. 1997, pp. 270–1; Davenport 2020). However, it is generally assumed that disease exposure was fairly uniform for rich and poor in this period, and so we might expect an urban penalty to manifest among wealthier occupational groups as well as poor ones.

The lack of any urban penalty among farmers and gentry may point to the importance of migration for the composition of urban populations. Gentry families were relatively mobile, and therefore gentlemen listed in rural and urban areas were equally likely to have spent at least part of their childhood elsewhere, and to have been exposed to some extent to urban disease environments. Indeed, this may explain the lack of any height advantage to gentlemen compared with farmers. Conversely, farmers who were listed in towns probably lived mainly in the rural portions of parishes containing towns. Therefore, we might expect relatively small differences between gentry and farmers in rural vs. urban parishes. However, poorer men in urban parishes were probably more likely to have been urban-born, or young single immigrants. This assumption is borne out by patterns of migration by social class in 1851. The 1851 Census was the first to record birthplace. In Dorset adult male labourers living in urban parishes were more likely than men in middle- and upper-class occupations to be born within 5 km of the centroid of their urban parish of residence (Day 2018). However, this difference was more marked for married men (57 per cent of labourers in urban parishes were locally born, compared with 31 per cent of middle- or upper-class men) than for single men (49 and 43 per cent, respectively). This is consistent with the observation in our sample that the steeper social gradient in height in urban parishes was observed only among married men. Thus to the extent that married urban labourers and craftsmen were more likely than wealthier men to have grown up in the town where they were listed, the urban penalty that they displayed may have reflected higher disease exposure in childhood.

Table 6 presents model results using the HISCLASS variable, stratified by marital status and urban/rural residence. Even when applying a very different social stratification scheme, we found clear social class gradients in adult height, especially for married men in urban parishes and single and married men in rural parishes. Unskilled workers were significantly shorter than the elite: among married men their height disadvantage was 2.9 cm in urban parishes and 2.4 cm in rural parishes. However, there was little distinction in height between skilled and unskilled workers (mainly labourers), despite relatively large differences in average wealth (Figure 6). As expected, there was no significant difference in height between the farmers and the elite (Table 6 and Figure 6).

**Table 6:** OLS regression results (dependent variable height in cm) using HISCLASS: single and married men in urban and rural parishes in Dorset, 1798–99

	Rural parishes		Urban parishes	
	Single men	Married men	Single men	Married men
*(Intercept)*	169.8	171.4	168.7	171.1
HISCLASS				
Elite	Ref.	Ref.	Ref.	Ref.
Lower middle class	−0.60	−2.01*	0.64	0.22
	*(0*.*95)*	*(0*.*91)*	*(0*.*97)*	*(0*.*93)*
Skilled workers	−1.65*	−1.78*	−0.93	−2.39**
	*(0*.*77)*	*(0*.*73)*	*(0*.*85)*	*(0*.*74)*
Farmers and yeomen	0.26	−0.51	1.24	0.23
	*(0*.*82)*	*(0*.*79)*	*(1*.*21)*	*(1*.*23)*
Unskilled workers	−1.62*	−2.38***	−1.01	−2.93***
	*(0*.*77)*	*(0*.*71)*	*(0*.*88)*	*(0*.*77)*
*R*^2^	0.02	0.02	0.02	0.05
Number of observations	2,877	2,274	933	669

***p < 0.001; **p < 0.01; *p < 0.05; †*p* < 0.1.

*Notes:* All models include control variables for health status and year of ballot. Ref. denotes the reference category. Figures in brackets are standard deviations.

*Source:* As for Table 2.

## Discussion

The results presented here using a representative sample of adult males for one English county from the turn of the nineteenth century support some of the previous findings based on military volunteers. English men were taller c. 1800 than European conscripts measured at any point before the late nineteenth century (Figure 1). Dorset men in our sample were on average 168.7 cm tall in 1798–99, and this tallies with estimates of older military volunteers in the same period (after adjustment for truncation; Floud et al. 1990, p. 148), suggesting that military data may provide a reliable guide to average heights for males in this period.

In contrast to the equivocal evidence for socio-economic differences in mortality in our period, our study found a clear gradient in stature by socio-economic status. However, this gradient was smaller than expected on the basis of the existing literature. It is often claimed that the wealthy could literally look down on the lower classes; however, the evidence for such large social class differences in height in England remains largely anecdotal (Floud et al. 1990, pp. 1–3). Floud et al. (1990) found relatively small differences in height between occupational groups among military volunteers in the nineteenth century. Despite the greater social breadth of our sample, we found similarly small differences. Labourers, the poorest men in English society in this period, were on average only 2 cm shorter than farmers and gentlemen (Figure 6). This degree of inequality in stature compares favourably with social class differences in height in the twentieth century (Floud et al. 1990, pp. 198–9; Li et al. 2004). The 1958 birth cohort study, for example, compared heights of males at age 33 according to the social class of their family during childhood. Men who were living in families classified as social classes 1 or 2 at age seven were on average 2.6 cm taller at age 33 than men who grew up in families that were classified as social classes 4 or 5 (Li et al. 2004).

Did we find only small differences in height by socio-economic status because our sample was restricted to Dorset, a relatively rural and healthy county? Although our sample lacked large towns, agricultural labourers were the poorest occupational group nationally (Lindert and Williamson 1983), and in Dorset labourers’ wages were low and housing probably poor even by national standards (Hunt 1986, pp. 965–6; Armstrong 1989, p. 744). In southern England, the late eighteenth century was a period of stagnant real wages, restructuring of the agrarian economy, and rising welfare costs as a result of underemployment, especially of agricultural labourers (Snell 1987, p. 151; Huzel 1989). A recent survey of autobiographical evidence from the period 1750–1850 concluded that it was largely the children of agricultural labourers and proto-industrial workers who were exposed to poverty-related hunger in the period—the children of urban industrial workers sometimes went hungry on account of feckless fathers, but not as a consequence of low wages (Griffin 2018). Although urban dwellers were often characterized as having a poor diet compared with rural populations, wages were higher in towns, and the English economy appears to have been well integrated with respect to food in this period (Humphries and Leunig 2009a).

Perhaps the most telling comparison in our sample was between farmers and agricultural labourers, that is, between the comfortably off, and the poorest men in rural parishes (Figure 6). Both groups would have grown up in fairly healthy environments (for the period). Indeed recent work has indicated that the children of agricultural labourers and of farmers experienced similar levels of mortality in childhood (Jaadla et al. 2020). However, agricultural labourers experienced poorer diets and possibly higher workloads during childhood, and this produced on average a 2 cm difference in adult height. In this sense, adult height revealed inequalities in childhood nutritional status that may not have been evident in mortality patterns*.* However, the extent of inequality detected by this measure was small.

Could the relatively small differences in average heights between gentry and farmers on the one hand and labourers on the other reflect a positive selection for height in physical occupations? It could be argued that agricultural labourers were selected for height and strength, especially compared with more sedentary occupations such as weaving, tailoring, or shoemaking (Kirby 1995). Indeed, labourers were on average taller than weavers and tailors despite the higher mean wealth (and higher HISCLASS rankings) of these latter occupations (Figure 6 and Table A2). However, we think such a selection effect is unlikely, for two reasons. First, height probably had relatively little effect on occupational mobility. Men could not become members of the gentry or farmers just because they were tall. And it is difficult to see why taller males would have been attracted into labouring work, given that labourers were the poorest occupational group in our sample (Figure 6).

Second, we found very little relationship between physical injury (which might be considered a more stringent selection criterion than height) and occupation. Among unmarried men (where a higher proportion of disabilities were probably of childhood origin and could have affected choice of occupation), labourers’ levels of physical injury were similar to those of tailors and shoemakers (4 per cent of males; Table A2). This is surprising. We might expect that some men were selected into sedentary occupations such as tailoring, shoemaking, or weaving on account of physical injury (either acquired in childhood and perhaps associated with stunting, or acquired in adulthood and necessitating a change of occupation). However, levels of physical disability were no higher than average among either married or unmarried tailors, shoemakers, and weavers (Table A2). On balance these results suggest that physical characteristics played a relatively minor role in choice of occupation among our sample. It should also be noted that our source reported habitual occupation rather than current employment status. Labouring was the default occupation for poor males in southern England in this period (40 per cent of all males in our sample). Underemployment was common, and therefore any effects of poor physical condition may have found expression in higher levels of underemployment or lower pay, rather than selection into (more highly paid) alternative activities.

If we accept the results reported here, then the question arises as to why there were such small differences in adult height of males by socio-economic status. Even privileged groups, such as the gentry and farmers, who should have been fairly well fed, were much shorter than modern populations, and not much taller than their servants and hired labourers. Two broad possibilities are suggested here. First, the disease environment may have been a major determinant of growth and attained height. This was a period when there appears to have been relatively little difference in longevity or childhood survival by wealth. Therefore, it is possible that the disease environment in childhood affected the growth of rich and poor fairly indiscriminately. This is consistent with the shorter stature of adult populations in even the very small towns in our sample.

A second possibility is that diet was the key determinant of height, and critical elements of diet were deficient for both rich and poor. While we might assume that the higher-status groups in our sample were reasonably well fed in childhood with respect to calories, the variety and seasonal availability of food remained limited for everyone. It is possible that the whole population was deficient in certain micronutrients, and some of these deficiencies may not have been completely remedied before the widespread fortification of food. It is also the case that the large disparities in wealth in English society were tempered to some extent by the Poor Laws, which operated in this period to maintain access to food for the poor during periods of dearth (Wrigley 2004, pp. 229–48). Indeed the relatively tall stature of English labourers, demonstrated here, lends some support to proponents of the view that English diets were fairly generous by international standards in this period (Muldrew 2011; Kelly and Ó Gráda 2013; Harris et al. 2015) and is consistent with arguments that England was a ‘high-wage’ economy (Allen 2015, 2019; for contrasting views see Humphries 2013; Stephenson 2019).

A further consideration with respect to socio-economic gradients in heights is the possibility that adult height is simply a rather insensitive measure of childhood development, because catch-up growth operates to smooth out much of the effects of nutritional deficits and disease episodes (Eveleth and Tanner 1990, pp. 190–6; Boersma and Wit 1997). Our sample included males aged 18–45, so most men in our sample had probably stopped growing. If men from wealthier families tended to cease growth earlier (or to experience an earlier adolescent growth spurt) than poorer men, then socio-economic differences in height would have been larger in childhood and early adulthood. In a rare longitudinal sample of Dutch recruits born 1790–1850, Beekink and Kok (2017) were able to compare heights of the same men at ages 19 and 24. They found that these men grew on average 5 cm between ages 19 and 24, and that socio-economic differences in height narrowed considerably over the same age range, because shorter males showed greater catch-up growth. If this phenomenon was widespread, then this could explain some of the variations in the magnitude of social class differences in heights in historical populations, because the average age at measurement varied between studies (see Table A1 in the supplementary material). It should be noted, though, that there was little evidence for such catch-up growth in our study, because socio-economic differences in height were very similar for married and single men. However, our sample of single men was older on average than most recruits.

Our study had a number of limitations. Most importantly, we lacked information on age or birth cohort. Our sample also omitted large towns, as already discussed. Additionally, our models assumed independence of observations; however, some men would have been from the same family, with shared genes that affected height. Our study was limited to the years 1798 and 1799, in the midst of war with revolutionary France, and it is possible that recruitment into the regular army affected the composition of our sample. We think this is unlikely, because most army recruits were young (aged 17–24) and unmarried (Floud et al. 1990, pp. 40, 60). However, to the extent that poorer males were recruited into the army, the height requirements imposed by the army probably acted to reduce the mean height of poorer men in our sample. That is, the effect of wartime should have been to exaggerate socio-economic differences in height, in our sample.

Despite these limitations, our study has provided a unique insight into the evolution of height inequalities in England, because it used the only known source of representative heights for males for the full wealth spectrum of English society before the twentieth century. While the differences in height of fully grown males by wealth or occupational status were small, they nonetheless suggested the existence of health inequalities in childhood. Whereas previous work has failed to find a disadvantage to children of labourers with respect to *survival*, it is clear that children who grew up to be labourers experienced poorer net nutritional status than the children of their employers and other higher-status social groups.

## Supplementary Material

Supplementary Material
