# Supplementary material for: Height and health in late eighteenth-century England
Source: Popul Stud (Camb). 2020 Sep 29;75(3):381–401. doi: 10.1080/00324728.2020.1823011 (PMC8516076; doi:10.1080/00324728.2020.1823011)
Supplement: Supplementary Material [file RPST_A_1823011_SM3315.pdf]

Supplementary appendices to Height and health in late eighteenth-century England. Hannaliis Jaadla, Leigh Shaw-Taylor and Romola Davenport. *Population Studies*. 2020

Table A1.

	Population	Data source	Period (collected)	Ages	Sample	Occupational groups compared	SES height difference	Mean height	Publication
1	Belgium	military (army) records	1816–1849	19	conscripts	Students – labourers (urban)	8.5	161.3	(Alter et al. 2004a)
2	Belgium	military (army) records	1850–1880	20	conscripts	Students – labourers (urban)	3.3	164.7	(Alter et al. 2004a)
3	Brazil	prison records	1861–1903	19–50	convicts	Professional – unskilled workers	3.2	164.3	(Baten et al., 2009)
4	Britain	military (army) records	1760–1820	21.6	volunteers	Agricultural workers – unoccupied	4.1	169.0	(Cinnirella 2008)
5	Britain	military (army) records	1820–1885	20.1	volunteers	Service workers – domestic workers	2.9	166.1	(Cinnirella 2008)
6	Britain	military (army) records	1914–1920	20.5*	volunteers	Middle class – unskilled workers	1.3	167.6	(Bailey et al. 2016)
7	Britain	military (army) records	c. 1837	17–24	volunteers	Merchants and vendors - servants	1.3	166.7	(Floud et al. 1990)
8	Canada	military (army) records	1899–1902	21–49	volunteers	Farmers – non-farmers	0.6	174.2	(Cranfield and Inwood 2007)
9	Canada	military (army) records	1914–1918	21–49	conscripts, volunteers	Farmers – non-farmers	1.2	172.0	(Cranfield and Inwood 2007)
10	Chile	military (army) records	1730–1800	17–55	conscripts	Landowners – others	2.2	167.4	(Llorca-Jaña et al. 2018)
11	France	military school records	1810–1826	14–23+	students	Students – conscripts	4.0	169.8	(Komlos 1994)
12	France	military (army) records	1816–1836	20.5*	conscripts	Farmers – agricultural workers	2.3	164.1	(Heyberger 2007)
13	Germany	school records	1771–1793	7–21	students	Aristocracy – lower class (age 20)	2.2	167.7	(Komlos 1990)
14	Germany	military (army) records	1834–1863	21	conscripts	White-collar – low status	3.2	167.9	(Lantzsch and Schuster 2009)
15	Germany	prison records	1856–1908	18–49	convicts	Middle class – others	1.4	167.2	(Baten & Murray, 2000)
16	Germany	military (army) records	1871–1913	21	conscripts	Upper class – working class	5.8	166.0	(Twarog 1997)
17	Italy	military (army) records	1764–1859	18–21	conscripts	Educated – textile workers	4.2	165.5	(A'Hearn 2003)
18	Italy	military (army) records	1856–1884	20	conscripts	White-collar – farmers	5.6	158.9	(Manfredini et al. 2013)
19	Italy	military (army) records	1886–1915	20	conscripts	Elite – unskilled workers	3.9	158.5	(Mazzoni et al. 2017)
20	Italy	military (army) records	1850–1890	20	conscripts	Construction/traders – farmers	4.6	163.3	(Manfredini et al. 2013)
21	Mexico	military (army) records	1850–1940	23+	volunteers	Skilled workers – unskilled workers	0.7	165.5	(López-Alonso and Vélez-Grajales 2015)
22	Mexico	passport records	1870–1920	18–50	passport holders	Elite – unskilled workers	2.5	170.3	(López-Alonso and Vélez-Grajales 2015)
23	Netherlands	military (army) records	1831–1861	19–20	conscripts	Others – unskilled workers	2.0	161.8	(Tassenaar 2019)
24	Netherlands	military (army) records	1850–1910	19	conscripts	Middle class – unskilled workers	2.0	168.5	(Quanjer and Kok 2019)

25	Netherlands	military (army) records	1815–1871	25	conscripts	Elite – unskilled workers	4.8	169.0	(Beekink and Kok 2017)
26	Spain	military (army) records	1879–1899	21	conscripts	Skilled workers – farm workers	2.9	163.3	(Ayuda and Puche-Gil 2014)
27	Spain	military (army) records	1866–1877	20	conscripts	Service workers – labourers (rural)	4.5	163.2	(Martínez-Carrión and Moreno-Lázaro 2007)
28	Spain	military (army) records	1866–1877	20	conscripts	Students – labourers (urban)	3.9	164.7	(Martínez-Carrión and Moreno-Lázaro 2007)
29	Spain	military (army) records	1895–1903	19	conscripts	Service workers – labourers (rural)	1.6	164.7	(Martínez-Carrión and Moreno-Lázaro 2007)
30	Spain	military (army) records	1895–1903	19	conscripts	Students – labourers (urban)	3.7	163.8	(Martínez-Carrión and Moreno-Lázaro 2007)
31	Sweden	military (army) records	1818–1881	20.9*	conscripts	White-collar – low skilled workers	4.0	164.0	(Öberg 2014)
32	Sweden	military (army) records	1881–1931	20.2*	conscripts	White-collar – low skilled workers	1.8	170.0	(Öberg 2014)
33	Switzerland	passport records	1813–1922	20–49	passport holders	Upper class – others	2.1	169.6	(Koepke et al. 2018)
34	Switzerland	prison records	1839–1931	20–49	convicts	Upper class – others	1.8	167.2	(Koepke et al. 2018)
35	Switzerland	military (army) records	1875–1884	19	conscripts	Upper class – lower class (urban)	4.2	164.5	(Schoch et al. 2012)
36	Switzerland	military (army) records	1885–1894	19	conscripts	Upper class – lower class (urban)	3.8	165.3	(Schoch et al. 2012)
37	Switzerland	military (army) records	1895–1904	19	conscripts	Upper class – lower class (urban)	4.4	166.1	(Schoch et al. 2012)
38	Switzerland	military (army) records	1905–1914	19	conscripts	Upper class – lower class (urban)	3.2	166.5	(Schoch et al. 2012)
39	USA	passport records	1830–1924	20–60	passport holders	White-collar – labourers	1.0	174.4	(Sunder 2013)
40	USA	military school records	1843–1894	16–21	cadets	Middle class – working class	1.2	171.8	(Komlos 1987)

Notes: * Mean age

Table A2. Height and health status by marital status in selected occupations, 1798–1799, and singulate mean age at marriage (SMAM) in 1851.

	SMAM (1851)	Mean height (SD)		#		Health status							
						Single men				Married men			
		Single men	Married men	Single men	Married men	No conditions	Physical injuries	Deaf, dumb or blind	Other	No conditions	Physical injuries	Deaf, dumb or blind	Other
Gentry	28.8	169.6 (4.1)	171.0 (4.1)	46	31	0.95	0.00	0.00	0.05	1.00	0.00	0.00	0.00
Farmers	25.0	170.1 (5.5)	171.0 (5.3)	362	213	0.95	0.04	0.01	0.00	0.94	0.02	0.02	0.02
Tailor	27.0	168.8 (6.6)	168.2 (5.2)	45	41	0.90	0.04	0.04	0.02	0.85	0.05	0.05	0.05
Shoemaker	26.2	168.0 (4.6)	169.0 (5.1)	26	23	0.88	0.04	0.04	0.04	1.00	0.00	0.00	0.00
Weaver	24.6	167.0 (4.9)	167.8 (5.4)	60	51	1.00	0.00	0.00	0.00	0.96	0.02	0.00	0.02
Servant	34.6	167.2 (6.2)	167.9 (6.8)	495	38	0.95	0.03	0.01	0.01	0.90	0.03	0.02	0.05
Labourers	26.7	168.0 (5.4)	168.7 (5.1)	1,433	1,319	0.93	0.04	0.02	0.01	0.91	0.06	0.02	0.01

Source: Medlycott 1999a; 1999b, own estimation.

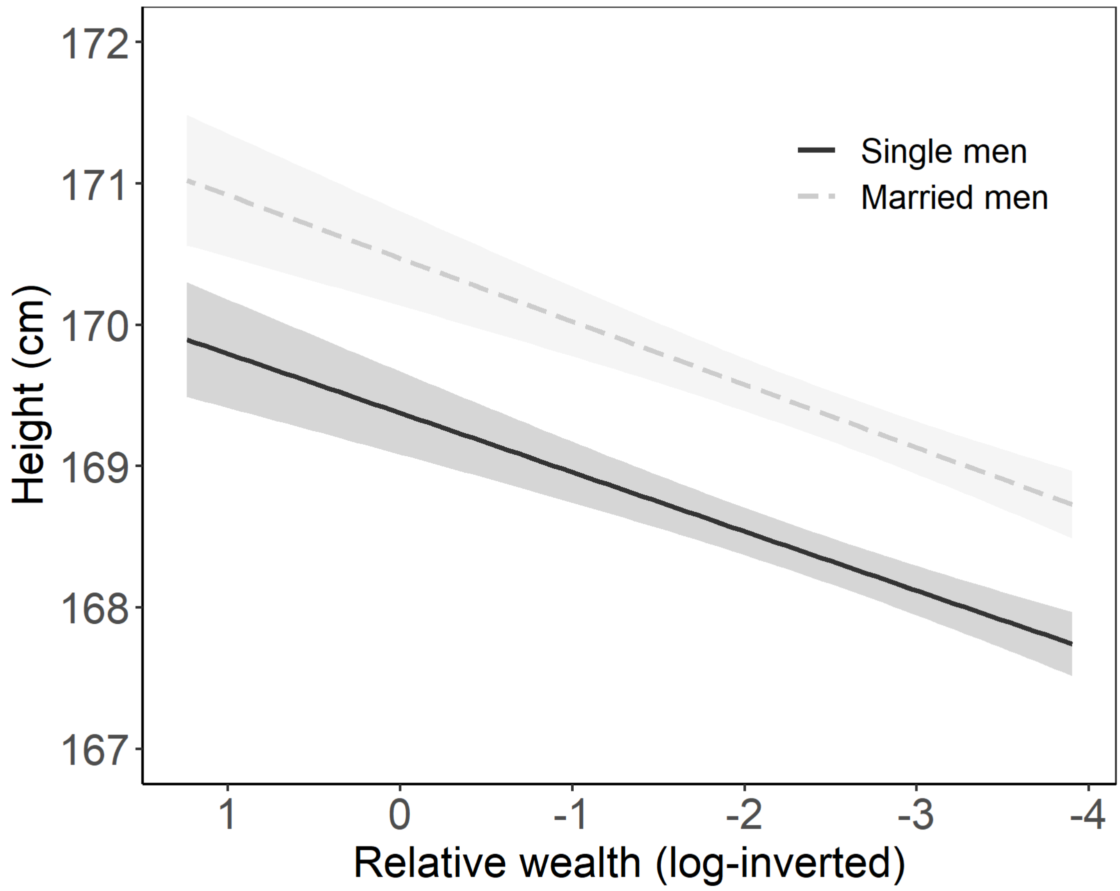

Figure A1. Predicted interaction effects of wealth and marital status on height, Dorset militia ballot lists, 1798–1799

Source: Medlycott 1999a; 1999b.
